# Effect of Intrathecal Bupivacaine Temperature Variation on Prophylaxis of Shivering in Urologic Patients Undergoing Spinal Anesthesia

**DOI:** 10.1155/anrp/1005074

**Published:** 2026-10-08

**Authors:** Emad M. Abdelhafez, Hala Gomaa, Sherif M. Abdelaziz, Esraa Sayed Ewais Muhammad, Heba Bakr

**Affiliations:** ^1^ Anesthesia, Surgical Intensive Care, and Pain Management Department Faculty of Medicine Cairo University Cairo Egypt cu.edu.eg; ^2^ Anesthesia, Surgical Intensive Care, and Pain Management Department Memorial Institute for Ophthalmic Research Giza Egypt

**Keywords:** anesthesia, bupivacaine, shivering, spinal, temperature, urologic surgical procedures

## Abstract

**Background:**

Postspinal shivering is a frequent complication of spinal anesthesia (SA) and is particularly relevant in urological procedures, where perioperative heat loss can be exacerbated by irrigation fluids. Modifying the temperature of the intrathecal local anesthetic may affect thermoregulatory responses and the properties of the blockade.

**Methods:**

This prospective, randomized, double‐blind, controlled trial was conducted in 96 patients (ASA I–II, aged 18–60 years) undergoing elective urological procedures under SA. Participants received intrathecal hyperbaric bupivacaine 0.5% (15 mg) combined with fentanyl 25 μg at one of three temperatures: 4°C (cold; *n* = 32), 24°C (room; *n* = 32), or 37°C (warm; *n* = 32). Shivering was graded using the validated Crossley and Mahajan scale. The primary endpoint was the incidence of clinically significant shivering (Grade ≥ 3) throughout the observation period, evaluated at 10‐min intervals.

**Results:**

Cold bupivacaine demonstrated a significantly higher incidence of clinically significant shivering (Grade ≥ 3) (81.3%) compared with the room‐temperature (34.4%) and warm (25%) groups (*p* < 0.001). The post hoc analysis showed that the difference between the room‐temperature and warm groups was not significant. Pharmacological intervention requirements were substantially elevated in the cold group (43.8%, 15.6%, 15.6%; *p* = 0.01). Warm bupivacaine accelerated sensory and motor block onset (*p* = 0.001) while reducing residual motor blockade.

**Conclusion:**

Warmed intrathecal bupivacaine significantly reduces PSS incidence and severity while optimizing block characteristics without compromising safety in urological patients.

**Trial Registration:** ClinicalTrials.gov identifier: NCT07081516.

## 1. Introduction

Postspinal shivering (PSS) is a significant complication of spinal anesthesia (SA), causing discomfort, anxiety, and physiological disturbances such as increased oxygen demand, hypoxemia, hypercapnia, elevated cardiac output, and lactic acidosis, especially in vulnerable patients [1]. Additionally, shivering compromises essential monitoring parameters, including blood pressure, electrocardiography, and oxygen saturation, thereby complicating intraoperative management and surgical conduct [2]. Urological patients encounter unique challenges, particularly exposure to room‐temperature irrigation fluids during transurethral surgeries, which may worsen perioperative hypothermia [3, 4]. Uncontrolled shivering during minimally invasive procedures may also impair surgical precision through involuntary patient movement, compromising delicate endoscopic maneuvers [5].

Endoscopic urological procedures were selected because many of these interventions require prolonged exposure to irrigation fluids, making patients particularly susceptible to perioperative heat loss and shivering. The temperature and volume of irrigation fluid can significantly affect perioperative thermal balance. Therefore, this population provides a clinically relevant setting in which to investigate the thermoregulatory effect of intrathecal injectate temperature while standardizing other important sources of perioperative thermal exposure [6, 7].

Management of perioperative shivering includes nonpharmacological strategies, such as warming intravenous [8, 9] and irrigation fluids [4, 7], forced‐air warming [10, 11], and maintenance of an appropriate operating‐room ambient temperature [12, 13].

Pharmacological strategies include clonidine [14], dexmedetomidine [15], ondansetron [15], midazolam [16], ketamine [17, 18], and tramadol [19, 20], along with nonpharmacological methods like warmed fluids, forced‐air warming [21], and optimal room temperature maintenance [22]. Pharmacological strategies have side effects (e.g., sedation, nausea, or hypotension), and warming alone may not offset irrigation‐related heat loss, highlighting the need for simple preventive measures. The long‐acting amide local anesthetic bupivacaine blocks voltage‐gated sodium channels, producing differential sensory‐motor blockade and a long duration of action [10]. Temperature affects bupivacaine’s solubility and diffusion characteristics [23]. Warming bupivacaine to 37°C reduces viscosity, facilitating more uniform subarachnoid dispersion, and may enhance neuronal penetration through an increased fraction of lipid‐soluble nonionized molecules [23, 24]. Warmed intrathecal bupivacaine may reduce PSS.

We hypothesized that variation in the temperature of intrathecal bupivacaine would influence the incidence and severity of PSS and the characteristics of spinal blockade without compromising safety. In urological populations receiving SA, the impact of varying the thermal properties of injected intrathecal bupivacaine on PSS prevention was assessed.

## 2. Patients and Methods

A total of 96 participants of both genders, aged 18–60 years and presenting with an American Society of Anesthesiologists (ASA) physical status of I or II, were recruited into this prospective, double‐blind, controlled, randomized trial. The cohort, which underwent planned urological interventions under SA, was evaluated across two Egyptian medical centers (Kasr Al‐Ainy Teaching Hospital and Students’ Hospital) over an eight‐month period spanning April 2025 to December 2025. The study protocol was approved by the Institutional Ethics Committee, and written informed consent was obtained from all participants, subsequent to the trial being sanctioned by the Research Committee (ID MS‐4‐2025) and registered at ClinicalTrials.gov entitled “Effect of Intrathecal Bupivacaine Temperature Variation on Prophylaxis of Shivering in Urologic Patients Undergoing Spinal Anesthesia.”

Patients were excluded if they refused a regional block, had neurological, psychological, hepatic, or renal disorders; had coagulopathy (defined as an INR greater than 1.4 and/or a platelet count less than 100,000/μL); had an infection or anatomical abnormality at the injection site; were hypersensitive to drugs; or their spinal blocks had either failed or were incomplete.

## 3. Methods and Group Allocation

This study was conducted and reported in accordance with the Consolidated Standards of Reporting Trials (CONSORT) guidelines for randomized controlled trials [25, 26]. Participants who underwent randomization were analyzed according to their originally assigned groups following the intention‐to‐treat principle. Utilizing computer‐generated sequences (https://www.randomizer.org/), the 96 suitable subjects were allocated equivalently across three cohorts (*n* = 32 per arm). To ensure concealment, these distribution codes were secured within sequentially numbered, sealed, opaque envelopes, which remained unopened until the conclusion of each individual’s prerandomization feasibility evaluation.

Group A (cold) received a bupivacaine‐fentanyl mixture stored at 4°C in a pharmaceutical refrigerator. Group B (room temperature) received the mixture maintained at 24°C via air‐conditioning adjustments. Group C (warm) received the mixture prewarmed to 37°C in a fluid‐warming cabinet.

### 3.1. Perioperative Management

A comprehensive medical history, physical examination, and laboratory investigations were performed for each patient. Special attention was paid to the spinal area to exclude local infection or anatomical deformities contraindicating neuraxial blockade.

Upon arrival at the operating room, preoperative monitoring was established, including continuous electrocardiography, pulse oximetry, noninvasive arterial blood pressure, and tympanic temperature; baseline vital signs were documented.

Intraoperative normothermia was maintained using a multimodal strategy. The operating room ambient temperature was set to 24°C, and all patients received a standardized preload of Ringer’s lactate warmed to 37°C at 10 mL/kg over the first 30 min, followed by a maintenance infusion of 6 mL/kg/h until completion of surgery. The replacement fluids, if needed, were also warmed to 37°C.

### 3.2. Blinding and Preparation

Ampoules were stored in coded bags at the designated temperatures. All study solutions were prepared under aseptic conditions by an independent, unblinded anesthesiologist who was not involved in patient management, outcome assessment, or statistical analysis. Immediately after preparation, each study solution was transferred to an identical sterile syringe and enclosed in an opaque insulated sleeve before being transferred to the attending anesthesiologist. The sleeve was used to minimize both visual and tactile temperature‐related cues, including condensation and differences in syringe surface temperature. The attending anesthesiologist administering the intrathecal injection, the patients, and the investigators responsible for outcome assessment remained blinded to treatment allocation. This procedure was used to conceal visual and tactile temperature‐related cues, including the appearance of condensation or differences in syringe temperature. Preparation and labeling were performed by personnel who were not involved in patient management, outcome assessment, or statistical analysis. SA was administered with the patient in the sitting position at the L4–L5 intervertebral space using a midline approach. The attending clinician, who remained blinded to the drug temperature, used a 25‐gauge Quincke spinal needle. Before patient enrolment, all blinded outcome assessors underwent standardized training in the application of the Crossley and Mahajan shivering scale. During the calibration phase, inter‐rater reliability was excellent, with an intraclass correlation coefficient (ICC) of 0.92. The same standardized assessment procedure was subsequently applied throughout the study.

All eligible patients underwent the institution’s routine preanesthesia feasibility assessment by the attending anesthesiologist before formal randomization. This assessment included evaluation of spinal anatomy, patient positioning, anticipated technical difficulty, and patient‐specific factors that could preclude timely administration of intrathecal anesthesia within the protocol‐defined 1‐min preparation‐to‐injection interval. Nine patients were considered unlikely to complete the preparation‐to‐injection process within this predefined interval and were therefore classified as prerandomization feasibility failures. These nine patients were excluded before randomization, and their allocation envelopes were not opened. Thus, no participants were excluded after randomization because of failure to meet the one‐minute preparation‐to‐injection interval. The feasibility assessment was applied uniformly to all screened participants before randomization.

Upon confirmation of successful intrathecal puncture and free cerebrospinal fluid (CSF) flow, the unblinded anesthesiologist prepared the intrathecal solution, comprising 15 mg of 0.5% hyperbaric bupivacaine (3 mL) and 25 μg of fentanyl. The mixture was maintained at the assigned nominal temperature before preparation and was immediately transferred into an identical sterile syringe. The syringe was then placed in an opaque insulated sleeve and promptly delivered to the blinded clinician. The interval from removal of the solution from its temperature‐controlled environment to completion of the subarachnoid injection was restricted to 1 minute to minimize temperature equilibration with the ambient environment [27, 28].

The protocol‐defined one‐minute interval referred to the time from removal/preparation of the study solution after successful intrathecal needle placement and confirmation of free CSF flow to completion of injection; it did not refer to the total duration of spinal needle placement or imply that all spinal procedures were successful on the first attempt.

Following successful injection, patients were immediately positioned supine.

“Sensory blockade was assessed using an ice pack applied bilaterally along the midclavicular line at 1‐min intervals. Sensory onset was defined as the time from the completion of the intrathecal injection to the complete loss of cold sensation at the T10 dermatomal level. This was determined by the patient’s inability to distinguish the cold stimulus from a neutral sensation when compared to a control area with intact sensation, such as the face or upper arm [29, 30].

The onset and maximum cephalad spread of sensory blockade were assessed at 0 (immediately after injection), 2, 4, 6, 8, and 10 min following intrathecal injection. The time of completion of the spinal injection was defined as 0 min.

Motor blockade was assessed at the same time points using the “Bromage scale [31]: [Grade 0: No motor block, Grade 1: Inability to raise extended leg, able to move knees and feet; Grade 2: Inability to raise extended leg and move knee, able to move feet; Grade 3: Complete motor block of the lower limbs.”

Sensory block onset was defined as the time required to reach the T10 dermatome, while motor block onset was defined as the time to achieve a Bromage score ≥ 2. Additionally, the maximum sensory block level at 10 min was recorded.

A failed block was defined as the absence of adequate sensory or motor blockade within 15–20 min following intrathecal injection, or a partial block requiring supplemental analgesia or conversion to general anesthesia. This 15–20‐min observation window ensured the timely identification of inadequate blocks, allowing for prompt intervention to maintain patient safety and optimal surgical conditions.

Hemodynamic parameters, including blood pressure, heart rate (HR), and oxygen saturation, were recorded at baseline before SA administration, at 5‐min intervals during the first 30 min after SA administration, and at 15‐min intervals until patient discharge from the postanesthesia care unit. Tympanic temperature was measured using an infrared aural (tympanic) thermometer (Granzia Auratemp Ear Thermometer, Italy) and was recorded at 20‐min intervals throughout the procedure.

To minimize confounding variables, thermal management was standardized: surgical drapes were used, room temperature was maintained at 24°C, and all intravenous and irrigation fluids were prewarmed to 37°C. Total irrigation volumes were recorded to ensure consistency across study arms.

The incidence of clinically significant PSS throughout the observation period was defined as a shivering score of ≥ 3 using the four‐grade scale described by Crossley and Mahajan [32]. Shivering was assessed at 10‐min intervals following SA. The grading system was as follows: “Grade 0, no shivering; Grade 1, one or more signs of piloerection, peripheral vasoconstriction, or peripheral cyanosis without visible muscle activity; Grade 2, visible muscle activity confined to a single muscle group; Grade 3, visible muscle activity involving more than one muscle group; and Grade 4, gross muscle activity involving the entire body. Grades 3 and 4 were considered clinically significant shivering and were additionally analyzed as a binary outcome (Grade ≥ 3)” [33].

We recorded the onset time of shivering following SA, maximum severity grade, and total episode duration.

Persistent Grade 3 or 4 shivering lasting more than 15 min was treated with pethidine 0.5 mg/kg intravenously. If shivering persisted after an additional 15 min or subsequently recurred, a supplemental pethidine dose of 0.25 mg/kg IV was administered [34]. The number of patients requiring primary and secondary interventions was documented.

### 3.3. Postoperative Management

A 24‐h postoperative follow‐up was conducted for every enrolled patient. In the postanesthesia care unit, motor blockade regression was assessed utilizing the Bromage scale at 10‐min intervals until complete recovery ensued; the timeframe from block initiation to the achievement of Bromage Grade 0 was defined as the motor blockade duration.

Postoperative pain intensity was evaluated using the “visual analog scale (VAS) [35], a validated 10‐cm linear scale ranging from 0 (no pain) to 10 (very severe pain)”. Assessments were conducted by the second anesthesiologist, who remained blinded to group allocation, at 1, 2, 4, 8, 12, and 24 h postoperatively. Patients reporting VAS scores exceeding 3 received rescue analgesia consisting of intravenous nalbuphine at 0.1 mg/kg, with a maximum single dose of 20 mg and a maximum daily dose of 160 mg.

Adverse events were systematically monitored and managed in accordance with standardized clinical protocols. Hypotension, defined as a reduction in MAP exceeding 20% from baseline, was treated with intravenous ephedrine at a dose of 5–10 mg. Bradycardia, characterized by an HR below 50 beats per minute, was managed with 0.5 mg of intravenous atropine. Pruritus, when present, was addressed with 25 mg of intravenous diphenhydramine. Episodes of nausea and vomiting were recorded, with moderate to severe cases treated using 4 mg of intravenous ondansetron.

The primary endpoint was the incidence of clinically significant PSS, defined as shivering of Grade ≥ 3 according to the Crossley and Mahajan scale [32], at any assessment during the observation period. Grades 3 and 4 were considered clinically significant shivering.

Secondary endpoints included maximum shivering severity, onset time and duration of shivering, requirement for pharmacological treatment, onset of sensory and motor blockade, maximum sensory block level, duration of motor blockade, perioperative hemodynamic and temperature changes, postoperative pain scores, rescue analgesic requirements, and adverse events.

## 4. Sample Size Calculation

Sample size was calculated a priori using G∗Power Software Version 3.1.9.2 (University of Kiel, Germany). The expected magnitude of the temperature‐related effect on shivering was informed by the randomized study of Najafianaraki et al. [28], which enrolled 78 parturients undergoing nonemergency cesarean delivery under SA. Participants received 10 mg of 0.5% hyperbaric bupivacaine stored either at 4°C or at approximately 23°C, with 10 μg of intrathecal fentanyl administered in both groups. The incidence of shivering was 39.1% in the cold‐bupivacaine group compared with 8.3% in the room‐temperature group. An omnibus chi‐square comparison across the three groups in the present trial was planned using an effect size (Cohen’s w) of 0.3914, a two‐sided *α* of 0.05%, and 90% statistical power. The resulting minimum total sample was 83 participants. To accommodate potential attrition and permit equal 1:1:1 allocation, the target sample was increased to 96 patients, with 32 patients assigned to each group.

### 4.1. Statistical Analysis

SPSS Version 26 (IBM Corp., Armonk, NY, USA) served as the primary analytical framework, initiating with Shapiro–Wilk and histogram assessments of data normality. Between‐group differences for normally distributed data (mean ± SD) were determined by one‐way ANOVA and Tukey’s post hoc testing, while nonparametric continuous data (median [Q1, Q3]) were analyzed using the Kruskal–Wallis test followed by Bonferroni‐corrected pairwise analyses. Categorical frequencies (%) were scrutinized with the chi‐square or Fisher’s exact test. To handle repeated postoperative observations of VAS scores, vital signs (HR, systolic and diastolic blood pressure), oxygen saturation, and temperature, GEE models with an unstructured correlation matrix were constructed to account for within‐patient correlations. Robust standard errors and Wald *χ* ^2^ statistics were utilized to test the significance of the main effects (group, time) and their interaction, with the interaction term specifically isolating variance in temporal trends across groups. The threshold for significance was set at a two‐tailed *p* < 0.05, applying Bonferroni corrections for multiple post hoc comparisons.

## 5. Results

A total of 122 patients were assessed for eligibility. Twenty‐six patients were excluded before randomization, including 11 who did not meet the eligibility criteria, six who declined participation, and nine who were identified during the preanesthesia feasibility assessment as unlikely to complete the preparation‐to‐injection process within the protocol‐defined one‐minute interval before randomization. Their allocation envelopes were not opened. The remaining 96 patients were randomized in a 1:1:1 ratio into three study groups (*n* = 32 per group). All randomized participants received their allocated intervention, achieved an adequate spinal block according to the prespecified criteria, completed follow‐up, and were included in the intention‐to‐treat analysis. No patient required conversion to general anesthesia because of failed or incomplete SA (Figure 1).

**FIGURE 1:**
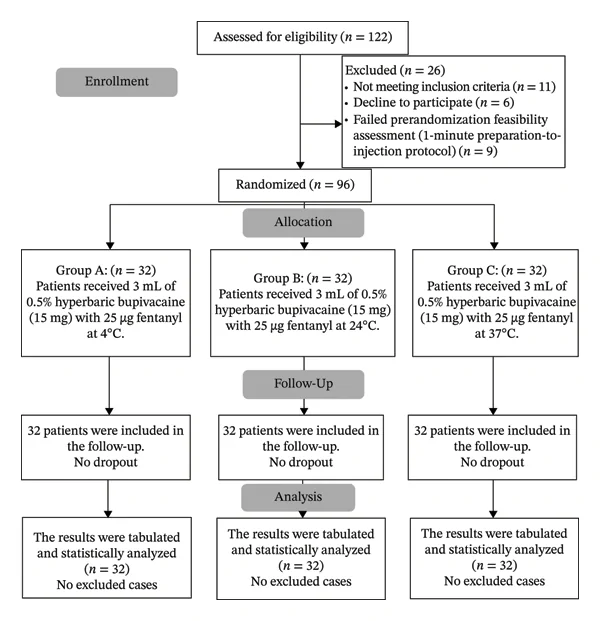
CONSORT flowchart showing study design.

Age, sex, ASA physical status, type of operation, associated comorbidities, and procedure duration were comparable among the three groups. There were no statistically significant differences between the three groups regarding the total volume of irrigation fluids administered during the procedures (*p* = 0.865), ensuring that external thermal exposure from irrigation was uniform across all study participants (Table 1).

**TABLE 1:** Demographic characteristics, comorbidities, and duration of procedure.

	Group A (*n* = 32)	Group B (*n* = 32)	Group C (*n* = 32)	*p*
Age (years)	44.50 ± 12.68	45.84 ± 13.38	41.59 ± 13.04	0.414
Sex				
Male	23 (71.9%)	28 (87.5%)	27 (84.4%)	0.238
Female	9 (28.1%)	4 (12.5%)	5 (15.6%)	
ASA physical status				
I	21 (65.6%)	17 (53.1%)	21 (65.6%)	0.495
II	11 (34.4%)	15 (46.9)	11 (34.4%)	
Type of operation				
TURP	9 (28.13%)	12 (37.5%)	7 (21.88%)	0.453
Ureteroscopy	16 (50%)	11 (34.38%)	13 (40.63%)	
Cystoscopy	7 (21.88%)	9 (28.13%)	12 (37.5%)	
Associated comorbidities				
Hypertension	4 (12.5%)	11 (34.4%)	5 (15.6%)	*X* ^2^ = 0.070
Diabetes mellitus	5 (15.6%)	7 (22.6%)	6 (18.8%)	*X* ^2^ = 0.815
Ischemic heart disease	2 (6.3%)	4 (12.5%)	2 (6.3%)	*X* ^2^ = 0.580
Chronic kidney disease (Stage I, II)	1 (3.1%)	1 (3.1%)	1 (3.1%)	*X* ^2^ = 1.000
Hypothyroidism	3 (9.4%)	1 (3.1%)	1 (3.1%)	*X* ^2^ = 0.430
Hepatitis C virus	1 (3.1%)	1 (3.1%)	1 (3.1%)	*X* ^2^ = 1.000
Duration of procedure (minutes)	106.25 ± 19.79	96.56 ± 28.52	90.78 ± 26.49	*F* = 0.051
Irrigation fluid volume (L)	**2.18 ± 0.52**	**2.25 ± 0.48**	**2.21 ± 0.55**	*F* = 0.865

*Note:* Data are expressed as mean ± SD or frequency (%). TURP: transurethral resection of the prostate, *F*: one‐way ANOVA, *χ* ^2^: chi‐square test.

Grade ≥ 3 shivering and pethidine use were significantly higher in Group A than in Groups B and C (*p* < 0.05), with no difference between B and C. Grade 4 shivering, tympanic temperature, second‐dose pethidine requirement, and disappearance time were comparable. Shivering onset occurred earlier in Group A than in B and C (*p* = 0.015), without difference between B and C. Sensory and motor block onset was progressively delayed from C to B to A (*p* < 0.05). Sensory level at 10 min and motor‐block duration were comparable (Table 2).

**TABLE 2:** Shivering data, onset, duration, maximum dermatomal block level, and time to first analgesic request.

	Group A (*n* = 32)	Group B (*n* = 32)	Group C (*n* = 32)	*p*	Post hoc
Incidence of shivering	26 (81.3%)	11 (34.4%)	8 (25%)	< 0.001^X2^	**P1 = 0.001**
					**P2 = 0.001**
					P3 = 0.412

Severity					
Grade 3	15 (46.9%)	5 (15.6%)	3 (9.4%)	**0.001** ^**X2**^	**P1 = 0.007**
					**P2** **<** **0.001**
					P3 = 0.449
Grade 4	11 (34.4%)	6 (18.8%)	5 (15.6%)	0.161^**X2**^	—

Onset of shivering (mins)	53.08 ± 21.41	31.36 ± 17.76	37.5 ± 22.04	**0.012** ^F^	**P1 = 0.015**
					P2 = 0.163
					P3 = 0.800

Tympanic temperature at the time of shivering (Celsius) (°C)	36.52 ± 0.07	36.52 ± 0.10	36.50 ± 0.05	0.839^F^	—

Pharmacologic methods (pethidine)	14 (43.8%)	5 (15.6%)	5 (15.6%)	**0.01** ^**X2**^	**P1 = 0.014**
					**P2 = 0.014**
					P3 = 1.00

Need for a second dose of pethidine	9 (28.1%)	3 (9.4%)	4 (12.5%)	0.098^**X2**^	—

Time of disappearance (min)	15.37 ± 5.53	16.50 ± 6.68	16.25 ± 6.94	0.855^F^	—

Onset of sensory block (mins)	7.0 ± 1.83	5.63 ± 1.62	4.50 ± 1.44	**0.001** ^F^	**P1 = 0.003**
					**P2 < 0.001**
					**P3 = 0.02**

Onset of motor block (mins)	6.81 ± 1.80	5.31 ± 1.59	4.13 ± 1.24	**0.001** ^F^	**P1 < 0.001**
					**P2 < 0.001**
					**P3 = 0.009**

Level of sensory block after 10 min	10 (8–12)	10 (10–10)	10 (8–10)	0.186^K^	—

Duration of motor block (time to Bromage Grade 0) (minutes)	125.63 ± 18.26	127.19 ± 13.79	124.06 ± 15.47	0.736^F^	—

*Note:* Data are expressed as mean ± SD, median [Q1, Q3], or frequency (%). P1: A versus B/C; P2: A versus C; P3: B versus C. F: One‐way ANOVA, *χ* ^2^: chi‐square test, K: Kruskal–Wallis test, post hoc: Bonferroni correction. Bold value means significant when *p* value < 0.05.

Incidence of shivering in all types of operation was significantly higher in Group A than in Group B and Group C (*p* value < 0.05) (Table 3).

**TABLE 3:** Subgroup analysis of the primary outcome according to operation type.

Incidence of shivering	Group A (*n* = 32)	Group B (*n* = 32)	Group C (*n* = 32)	*p* value
TURP	6/9 (66.7%)	3/12 (25.0%)	0/7 (0.0%)	**0.012**
Ureteroscopy	13/16 (81.3%)	4/11 (36.4%)	5/13 (38.5%)	**0.028**
Cystoscopy	7/7 (100%)	4/9 (44.4%)	3/12 (25.0%)	**0.004**

*Note:* Data are expressed as frequency (%). TURP: transurethral resection of the prostate. Test used was chi‐square test. Bold value means significant when *p* value < 0.05.

Generalized estimating equations analysis demonstrated significant effects of treatment group (Wald *χ* ^2^ = 10.000, df = 2, *p* = 0.007), time (Wald *χ* ^2^ = 83.295, df = 5, *p* < 0.001), and the group × time interaction (Wald *χ* ^2^ = 42.067, df = 10, *p* < 0.001) on postoperative VAS scores. These findings indicate that postoperative pain changed significantly over time and that the temporal pattern of VAS scores differed significantly among the three study groups (Table 4).

**TABLE 4:** Generalized estimating equations analysis of postoperative VAS scores, vital signs, and temperature.

Source		Wald *χ* ^2^	df	*p* value
VAS scores	Group	10.000	2	**0.007**
	Time	83.295	5	**< 0.001**
	Group × time	42.067	10	**< 0.001**

SPO_2_	Group	2.289	2	0.318
	Time	154.888	12	**< 0.001**
	Group × time	66.673	24	**< 0.001**

Heart rate	Group	1.796	2	0.407
	Time	159.778	12	**< 0.001**
	Group × time	75.870	24	**< 0.001**

Temperature	Group	8.460	2	**0.015**
	Time	70.860	5	**< 0.001**
	Group × time	26.659	10	**0.003**

MAP	Group	4.705	2	0.095
	Time	457.784	12	**< 0.001**
	Group × time	63.379	24	**< 0.001**

*Note:* SpO_2_: peripheral oxygen saturation. VAS scores, vital signs, and temperature were analyzed using generalized estimating equations (GEEs) with treatment group, time, and the group × time interaction included as fixed effects. An unstructured working correlation matrix and robust standard errors were used to account for within‐subject correlation. Statistical significance was set at *p* < 0.05. Bold value means significant when *p* value < 0.05.

Abbreviations: MAP = mean arterial pressure, VAS = visual analog scale.

Generalized estimating equations analysis showed no significant overall differences among the three groups in either SpO_2_ (Wald *χ* ^2^ = 2.289, df = 2, *p* = 0.318) or HR (Wald *χ* ^2^ = 1.796, df = 2, *p* = 0.407). However, both parameters changed significantly over time (SpO_2_: Wald *χ* ^2^ = 154.888, df = 12, *p* < 0.001; HR: Wald *χ* ^2^ = 159.778, df = 12, *p* < 0.001) and demonstrated significant group‐by‐time interactions (SpO_2_: Wald *χ* ^2^ = 66.673, df = 24, *p* < 0.001; HR: Wald *χ* ^2^ = 75.870, df = 24, *p* < 0.001), indicating that the temporal patterns of oxygen saturation and HR differed significantly among the three study groups. Generalized estimating equations analysis demonstrated significant effects of treatment group (Wald *χ* ^2^ = 8.460, df = 2, *p* = 0.015), time (Wald *χ* ^2^ = 70.860, df = 5, *p* < 0.001), and the group‐by‐time interaction (Wald *χ* ^2^ = 26.659, df = 10, *p* = 0.003) on perioperative temperature. The significant interaction indicated that the pattern of temperature change over time differed significantly among the three study groups. Table 4.

A significant difference was found in VAS scores at 2 and 4 h postoperatively between Group A and Group B (*p* = 0.001 and 0.009, respectively), while no significant difference was found between Group C and Groups A and B. No significant difference was observed at 1, 8, 12, and 24 h among the three groups. Nalbuphine use was greatest in B (34.4%), A (28.1%), and least in C (6.3%), with a significant difference between Group C and Groups A and B (Table 5).

**TABLE 5:** VAS score at the time of measurement (over 24 h) and the need for nalbuphine between groups.

		Group A (*n* = 32)	Group B (*n* = 32)	Group C (*n* = 32)	*p*	Post hoc
VAS score	First hour	0 [0,0]	0 [0, 0]	0 [0, 0]	0.33^K^	—
	2^nd^ hour	0 [0,0]	2 [0, 4.25]^B^	0 [0, 1]^C^	**0.006** ^K^	**P1 = 0.001** P2 = 0.095 P3 = 0.131
	4^th^ hour	0 [0, 4]^A^	4 [0, 8]^B^	0.5 [0, 2]^C^	**0.004** ^K^	**P1 = 0.009** P2 = 0.656 P3 = 0.002
	8^th^ hour	3 [0, 5]^A^	3 [0, 5]^B^	1 [0, 2.25]^C^	0.082^K^	—
	12^th^ hour	2 [0, 5]^A^	0 [0, 3.25]^B^	0.5 [0, 2]^C^	0.140^K^	—
	24^th^ hour	0 [0, 3.25]^A^	0 [0, 2]	0 [0, 1.2]^C^	0.580^K^	—
Number of patients asked for nalbuphine (%)		9 (28.1%)	11 (34.4%)	2 (6.3%)	**0.019** ^**X2**^	P1 = 0.589 **P2 = 0.020** **P3 = 0.005**

		***n* = 9**	***n* = 11**	***n* = 2**		

Number of patients asked for nalbuphine	At 2^nd^ hour	3 (33.3%)	1 (9.1%)	1 (50%)	**0.008** ^**X2**^	—
	At 4^th^ hour	3 (33.3%)	9 (81.8%)	0 (0%)		
	At 5^th^ hour	0 (0%)	0 (0%)	1 (50%)		
	At 8^th^ hour	3 (33.3%)	1 (9.1%)	0 (0%)		

*Note:* Data are expressed as median [Q1, Q3] or frequency (%). A: significantly different compared with baseline assessed for Group A, B: significantly different compared with baseline assessed for Group B, C: significantly different compared with baseline assessed for Group C. P1: A versus B/C; P2: A versus C; P3: B versus C. X2: chi‐square test, K: Kruskal–Wallis test, post hoc: Bonferroni correction (represents comparison of the overall distribution of requests between groups and a comparison at specific time points). Bold values means significant when *p* value < 0.05.

Bradycardia occurred in 9.4% of Group A, with none in Groups B or C (significant difference). No significant differences were noted for hypotension, nausea, and vomiting. Pruritus occurred in 4 patients (12.5%) in Group A, 7 patients (21.9%) in Group B, and 2 patients (6.3%) in Group C, with no statistically significant difference among the groups (*p* = 0.184) (Table 6).

**TABLE 6:** Incidence of postspinal adverse effects between groups.

	Group A (*n* = 32)	Group B (*n* = 32)	Group C (*n* = 32)	*p*
Hypotension	10 (31.3%)	10 (31.3%)	4 (12.5%)	0.135^**X2**^
Bradycardia	3 (9.4%)	0 (0%)	0 (0%)	**0.045** ^**X2**^
Nausea and vomiting	5 (15.6%)	7 (21.9%)	4 (12.5%)	0.59^**X2**^
Pruritus	4 (12.5%)	7 (21.9%)	2 (6.3%)	0.184^**X2**^

*Note:* Data are expressed as frequency (%). *χ* ^2^: chi‐square test. Bold value means significant when *p* value < 0.05.

A significant difference in systolic blood pressure appeared only at 10 min. Diastolic pressure showed significant differences at 10, 15, and 20 min. No significant variations were observed among groups regarding SpO_2_ or HR across all measured time points. A significant temperature difference appeared only at 60 and 80 min (Figure 2).

**FIGURE 2:**
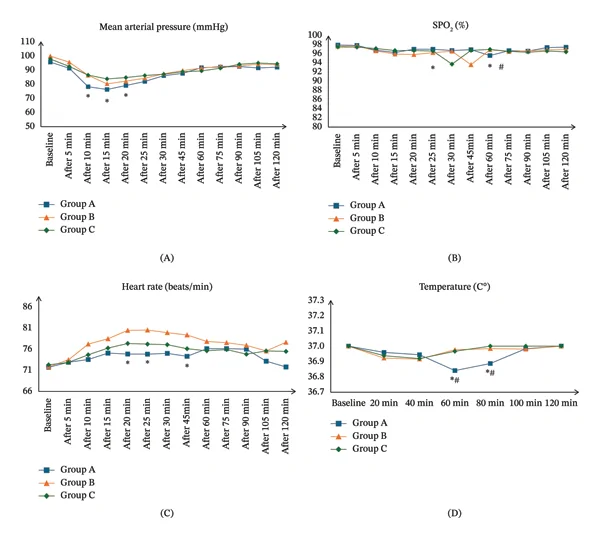
(A) Mean arterial pressure, (B) SpO_2_, (C) heart rate, and (D) temperature changes.

## 6. Discussion

The primary objective of this study was to evaluate the influence of intrathecal bupivacaine temperature on shivering prophylaxis and block characteristics in urological patients. Our results demonstrate that a cold local anesthetic mixture to 4°C significantly increased the incidence and severity of PSS. Specifically, we observed a shivering incidence of only 25% in the warm group compared to 81.3% in the cold group (*p* < 0.001), which directly correlates with a reduced requirement for rescue pethidine (15.6% vs. 43.8%).

The pathophysiology of PSS involves a complex interplay of central and peripheral factors [28]. SA typically redistributes core heat to the peripheral tissues and impairs hypothalamic thermoregulatory control [2]. However, the injection of a cold solution (4°C) into the subarachnoid space may independently trigger shivering by activating spinal thermosensors, even before a significant drop in core body temperature is recorded [36, 37]. Our finding that shivering onset occurred significantly earlier in the cold group (53.08 ± 21.41 min) compared to room‐temperature and warm groups suggests that the “thermal shock” of the injectate acts as an immediate trigger for the shivering reflex [27, 28]. Warming the bupivacaine to 37°C mitigates this trigger, thereby elevating the shivering threshold and providing effective prophylaxis [28, 37].

Beyond its thermoregulatory effects, injectate temperature significantly alters the physical properties of bupivacaine. Warming the hyperbaric solution to 37°C reduces its viscosity, which likely facilitates more uniform subarachnoid dispersion and rapid neural penetration [38]. This mechanism explains the significantly faster sensory onset (4.50 ± 1.44 min) and motor onset (4.13 ± 1.24 min) observed in our warm cohort compared to the cold group [27, 38]. While some literature suggests that high baricity is the primary determinant of bupivacaine spread [39], our data align with the findings of Zhao et al. [24] noted that warming enhances the clinical potency and distribution efficiency of the drug.

Urological surgery provides a particularly relevant clinical model for evaluating temperature‐related interventions because endoscopic procedures commonly require irrigation, which represents an additional source of perioperative thermal exchange. Previous evidence has demonstrated that warming irrigation fluids reduces perioperative hypothermia and shivering. In the present study, irrigation fluids were therefore standardized at 37°C and their total volumes were recorded in an attempt to minimize this important source of confounding [4, 24]. The uniformity of irrigation volumes across our study groups (*p* = 0.865) ensures that the observed differences in shivering were a direct result of the intrathecal temperature variation rather than external thermal exposure [27, 28].

Our results are broadly consistent with studies by Kumar et al. [40] and Henriques et al. [27] who reported that warming intrathecal solutions to body temperature effectively mitigates shivering compared to room‐temperature alternatives.

Similarly, Al‐Mandhari et al. [39] observed marked reductions in shivering when using body‐temperature heavy bupivacaine. In contrast, Kishore et al. [41] found no significant difference in shivering incidence across temperatures, a discrepancy that may be attributed to variations in patient demographics, anesthetic techniques, or the coadministration of different adjuvants.

### 6.1. Limitations and Clinical Implications

A notable limitation of this study is the universal administration of intrathecal fentanyl across all groups, which likely introduced a pharmacological “ceiling effect” on shivering prophylaxis [42]. This interaction may have blunted the overall shivering response, potentially masking more significant differences between the warm and room‐temperature cohorts and underestimating the true clinical impact of bupivacaine warming. Furthermore, while infrared tympanic thermometry is practical for awake patients, it may lack the precision of gold‐standard invasive monitoring of core temperature [43]. This could lead to an underestimation of the absolute degree of perioperative hypothermia or a failure to detect rapid, subtle core temperature fluctuations that trigger the shivering reflex [44]. In addition, although visual and tactile temperature‐related cues were minimized by transferring all study syringes in identical opaque insulated sleeves, the success of blinding was not formally assessed. Therefore, subtle temperature‐related cues and consequent residual unmasking cannot be completely excluded. Although irrigation‐fluid temperature was standardized and irrigation volume and procedure duration were recorded, predefined upper limits for procedure duration and total irrigation volume were not used as eligibility criteria. Residual variability in cumulative thermal exposure may therefore remain. Future studies should incorporate a sterile in‐line thermocouple or equivalent validated temperature‐monitoring device to confirm the injectate temperature immediately before intrathecal administration.

## 7. Conclusions

Cold bupivacaine (4°C) increases the incidence and severity of PSS in urologic patients compared to room‐temperature (24°C) solutions and warm (37°C) bupivacaine. Warming intrathecal bupivacaine to 37°C accelerates sensory and motor block onset and improves early postoperative analgesia without increasing adverse events.

## Funding

The authors have nothing to report.

## Conflicts of Interest

The authors declare no conflicts of interest.

## Data Availability

Data are available from the authors upon request.
